# Reshaping the resilient educational ecosystem in the intelligent era: a case study of the CLIL course of international trade practice

**DOI:** 10.3389/fpsyg.2026.1807137

**Published:** 2026-04-16

**Authors:** Xiaoqin Yin, Fang Chen

**Affiliations:** 1 School of Foreign Languages, Hangzhou City University, Hangzhou, Zhejiang, China; 2 College of Education, Zhejiang University, Hangzhou, Zhejiang, China

**Keywords:** CLIL, intelligent era, International Trade Practice, resilient educational ecosystem, smart course

## Abstract

This study explores the transformation of the educational ecosystem of the CLIL course of International Trade Practice in the intelligent era, with the goal of constructing an interdisciplinary resilient educational ecosystem that adapts to the development of artificial intelligence. We have proposed a resilient educational ecosystem paradigm for International Trade Practice that features an AI-empowered ecological learning environment, the collaborative dynamic interaction among teacher-student-intelligence, the integration of adaptive large-scale teaching and individualized teaching, and a holographic smart evaluation method. A quasi-experimental control group design is employed to assess the teaching effectiveness of the resilient educational ecosystem in the International Trade Practice course at Hangzhou City University. Quantitative results indicate that the teaching reform has exerted significant impacts on enhancing students’ academic performance (*p* < 0.01, *d* = 0.988). Specifically, although no statistically significant differences were found in multiple choice question (*p* < 0.996) and calculation (*p* < 0.988), the experimental group performed significantly better than the control group in terminology translation (*p* < 0.01, *d* = 0.944), case analysis (*p* < 0.05, *d* = 1.002), and documentation question (*p* < 0.01, *d* = 0.876). The findings reinforce the specific functions of a resilient educational ecosystem in enhancing higher-order cognitive abilities like analyzing real cases, thus providing empirical substantiation for subsequent optimization in the intelligent era. A resilient educational ecosystem improves students’ comprehensive qualities, promotes educational equity, narrows educational gaps between universities and regions, and promotes lifelong learning. It adapts to the development needs of future society and has important enlightenment for global education reform and development.

## 1 Introduction

Digital-intelligent transformation serves as a significant vehicle and future vision for the world education transformation in the era of artificial intelligence (AI). AI technology has reshaped the ecological landscape of educational reform. It is imperative to establish a teaching paradigm that aligns with the demands of the intelligent era. The World Digital Education Conference in 2024 released the AI Empowering Education Initiative, which posits that AI will serve as a pivotal driver for advancing high-quality education, fostering innovative paradigms and new forms of learning (Zhou et al., 2025).

As an emerging concept in the field of educational technology, resilient educational ecology combines system resilience theory with AI technology support for the purpose of ensuring continuity, fairness, and high-quality educational activities in uncertain environments. With the development of digital intelligent technology and the evolution of educational policies, China’s educational ecological structure is being reshaped, necessitating the adherence to innovative teaching concepts and methods to cultivate diverse talents that meet contemporary demands. Exploring the construction of CLIL (content and language integrated learning) educational ecological pathways in the context of International Trade Practice and evaluating their effectiveness in establishing a healthy, coordinated, and orderly “discipline + foreign language” interdisciplinary intelligent educational ecosystem is an important research topic in Chinese educational ecology. However, existing research still lacks specific practical cases to clarify the manifestations and teaching paradigms of intelligence empowered educational ecosystem in CLIL courses in China. This research intends to explore the construction of such an ecosystem and evaluate its teaching effectiveness, hoping to provide theoretical and practical references for the reform and innovation of international trade education. The research will help enrich CLIL theory because it addresses the current over-reliance on foreign language teaching globally, which lacks compatibility with interdisciplinary courses in CLIL theory. It will also expand digital-intelligent educational theory by demonstrating how AI technology functions as a empowering element in educational ecosystem, thus providing theoretical support for its deep integration with interdisciplinary teaching.

The subsequent sections of this paper are organized as follows: The study begins with a brief literature review, followed by the construction of a paradigm for a resilient educational ecosystem. Subsequently, a quasi-experimental control group design is utilized to compare the teaching efficacy of the resilient education ecosystem with that of the traditional teaching model in the International Trade Practice course at Hangzhou City University. Finally, the study concludes by summarizing the research findings, discussing the implications for educational practice, and proposing suggestions for future research endeavors.

## 2 Literature review

### 2.1 International trade practice course

International trade serves as a primary indicator of openness, making a significant contribution to the country’s economic growth and prosperity (Abadiyah and Endraswati, 2023; Titus et al., 2022). The evolving trends in society, economy, science, technology, and culture in the 21st century, coupled with the challenges of “trade war” and “de-globalization” to higher education (Zhao et al., 2025), have led to continual shifts in China’s demand for diverse talents. Simultaneously, the criteria for talent qualifications have been continuously rising. Taken together, these factors make continuous reform in higher education essential (Lan, 2022).

International Trade Practice is a core course for the Business English major at application-oriented universities. The course teaches students about the entire performance procedures of handling import and export contracts while providing English language instruction. The traditional educational system faces multiple difficulties such as fragmented knowledge, identical and large-scale teachings, and monolithic evaluation, which have fallen behind the progress of the discipline of intentional trade (Zhu, 2021). The course consists of various learning segments which cover trade terms and transportation insurance and payment methods for goods and services. The knowledge points within these modules are scattered, and there is a lack of systematic correlation among the modules. The current educational system blocks students from developing complete knowledge frameworks which forces them to concentrate on details rather than the overall picture. Moreover, the teaching content chosen by different institutions are confined to their access to the educational resources and teachers’ professional competence and digital literacy. It fails to incorporate the industry trends, regional economic characteristics, and students’ requirements into the course planning process, making it challenging to engage students and meet their diverse career development needs. The current teaching model predominantly depends on in-class lectures delivered by teachers. This “cramming” teaching approach is widespread, leading students to passively gain knowledge. They have few opportunities for active engagement, practical operation, and in-depth contemplation. There is a lack of interaction and experiential learning, which does not align with modern educational ideas. The course evaluation exhibits an over-reliance on summative evaluation approaches such as final examinations. This approach emphasizes knowledge retention, yet overlooks comprehensive evaluations of language application proficiency, cross-cultural communication capabilities, problem-solving skills, and creative thinking abilities. Thus, it fails to truly reflect the students’ learning achievements and course quality. To address the issues above mentioned, it is imperative to explore novel teaching paradigms to invigorate the International Trade Practice course, thereby enhancing the quality of teaching and the comprehensive competitiveness of students.

### 2.2 CLIL

CLIL represents a crucial educational strategy and model that European Union countries have extensively adopted since the 1990s. It synthesizes experiences from diverse models such as total immersion education, bilingual education, content-based education, and cross-curricular English learning. This approach demonstrates a transformation in teaching philosophy from a skill-centered orientation to a content-reliant approach, and further progresses towards the integration of content and language. Scholars view CLIL as a catalyst for reforming language education. The research hotspots and frontier themes in this area, though diverse, exhibit consistent alignment. In its early phases, CLIL research primarily focused on how this educational approach fosters general foreign language acquisition. Besides theoretical introductions (Sheng, 2015; Liu and Huang, 2013), mainstream research focuses on CLIL’s positive impact on learning motivation (Coyle et al., 2010; Lasagabaster, 2011; Lo, 2024), affective factors (Yuan, 2012; Jiang et al., 2022), foreign language proficiency (Coyle, 2007; Coyle et al., 2010; Dalton-Puffer, 2011; Dalton-Puffer, 2013; Dalton-Puffer, 2016; Dalton-Puffer and Smit, 2013; Chang et al., 2009; Zhang and Pladevall-Ballester, 2021; Sato, 2024). Nevertheless, in recent years, the collaborative development of language, critical thinking, and disciplinary competence (Dallinger et al., 2016; Wu and Chang, 2011; Zhang and Li, 2019; Zhao et al., 2025; Song et al., 2023; Salvador-García et al., 2020; Bower et al., 2020; Jiang et al., 2022; Bermejo et al., 2021; Bekirogulları et al., 2022; Dalton-Puffer et al., 2024), the technology integrated CLIL programs(Hu et al., 2025), and the professional development and awareness of CLIL teachers (Hemmi and Banegas, 2021; Li, 2024), and the design of CLIL course materials (Ting, 2024), especially as reflected in the utilization of disciplinary language, have become increasingly conspicuous in CLIL research. Studies have shown that CLIL significantly enhances receptive language skills, enriches productive vocabulary breadth and fluency, raises pragmatic awareness, fosters positive learning emotions, and promotes the integration of language and cognition along with the collaborative development of disciplinary knowledge. However, prior studies indicated that students experienced the challenge of learning subject content in a foreign language (Lee et al., 2025; Roussel et al., 2017). Existing studies predominantly focus on the instruction of language courses through content integration, the impacts of CLIL on the acquisition of content knowledge “remain inconclusive and less promising” (Hu et al., 2025). The effectiveness of language teaching does not signify the overall teaching effectiveness. It is necessary to conduct further exploration into the application and teaching efficacy of CLIL in non-foreign language subjects.

The Declaration on the Construction of New Liberal Arts underscores that education must expedite innovative development, necessitating the establishment of new classrooms from the perspectives of both enhancing content quality and innovating teaching models (Wu, 2020). Model innovation is reflected in aspects such as teaching content, teaching methods, and means (Dai et al., 2020). In the context of digital intelligence in higher education, exploring the innovative paradigms for New Liberal Arts CLIL and evaluating its effectiveness bear both theoretical and practical implications.

### 2.3 Intelligence empowered resilient educational ecosystem

Since 2018, the acceleration of industrial transformation and the flourishing development of AI have gradually ushered China into a new era of smart education. Themes like “reshaping a learner-centered new educational ecosystem”, “building a smart education ecosystem”, and “reconstructing a healthy educational ecology” have started to gain increasing attention (Li and Yue, 2025). In the era of AI, the educational ecosystem exhibits the following intelligent characteristics: teaching methodologies are evolving towards human-AI cooperation and automation, learning is progressing towards individualization, customization, and lifelong learning, management is advancing towards intelligent administration with inventive teamwork, and evaluation is expanding towards enhanced accuracy, immediacy, and thoroughness (Tang and He, 2019). AI reshapes the resilient educational ecosystem from two dimensions of system ontology and technological applications. A resilient educational system possesses the ability to self-adjust and transform, can withstand external impacts, and maintain continuous operation. Digital intelligence technology impacts the subjects, content, activities, and environment of the educational ecosystem, thereby triggering systemic changes and promoting the development of teaching towards data-driven directions to achieve effective adaptation in complex environments (Tang and He, 2019). “China Education Modernization 2035” (Ministry of Education, 2019) focuses on the construction of educational informatization and presents new requirements for the integration of technology and learning environment design: achieving human-machine interaction at the teaching level, shifting towards personalized, customized, and lifelong learning domains, advancing governance towards intelligent management models, expanding evaluation dimensions towards high precision, real-time and comprehensive assessments, and moving the environmental situation towards a multidimensional space that combines virtual and reality (Tang and He, 2019).

The resilient, intelligent educational ecosystem, characterized by digitization, integration, interaction, and diversity, has become the evolutionary trend in current higher education teaching systems. Researchers have shifted from the “static capability” perspective to the “dynamic process-system-technology” integration perspective to explain the intelligent educational ecosystem. Scholars precisely push learning resources with intelligent tutoring systems to solve students’ personalized learning issues (Wang and Dan, 2020), mine data using machine learning algorithms to construct learner profiles and empower teachers’ teaching (Zhai et al., 2021), construct situational classrooms with intelligent virtual reality technology and provide real-time feedback on student learning status through human-machine interaction technology (Kelly et al., 2018), create human-machine collaborative teaching models based on digital intelligence technology to realize pre-class diagnosis, in-class aided learning, and post-class evaluation (Gao et al., 2021), disclose the key factors affecting teachers’ behavioral intentions in the AI education ecosystem (Wu et al., 2025) and elucidate the interrelationship of various ecological factors in the ecological environment of college mathematics classrooms under the internet environment (Han, 2024). However, existing studies that focus specifically on the construction of a resilient educational ecosystem for interdisciplinary CLIL courses in International Trade Practice remain relatively limited. Given China’s prominent status as one of the world’s top trading nations, it becomes increasingly crucial to systematically examine the pathways for building such an ecosystem and to rigorously evaluate its effectiveness. This line of research is essential for cultivating high-quality interdisciplinary foreign language talents equipped to thrive in the intelligent era.

## 3 The construction of a resilient educational ecological paradigm for the course of international trade practice

The emergence of the ecological paradigm is attributed to advancements in the natural sciences, education within this paradigm should be conceptualized and executed in alignment with the principles and attributes inherent in ecological systems. The educational transformation of constructing a resilient educational ecosystem taking place at this in-between, and in-process moment, represents a unique time in our educational ecology, where new meets old, questions of values and resources are being reconsidered, and stakeholders are not yet fully adapted to what the future will bring (Edwards and Magill, 2023). Paradigm construction is the primary issue in creating the resilient educational ecosystem empowered by AI (Tang and He, 2019). The course paradigm should primarily tackle the pedagogical dilemmas inherent to that discipline, while pursuing technological assistance. We built a resilient educational ecosystem for International Trade Practice characterized by the AI empowered ecological learning environment, the collaborative dynamic interaction among teacher-student-intelligence, the integration of large-scale and adaptive individualized teaching, and holographic smart evaluation method (Zhu and Peng, 2020). By innovating teaching methods, enriching teaching resources, and fully utilizing intelligence for course content construction, precise pushing and feedback, and evaluation, we can provide more personalized, efficient, and high-quality educational services (Zhang and Andrés, 2024) for students enrolled in the course of International Trade Practice.

The resilience of this educational ecosystem is defined by its absorptive, adaptive, and transformative capacities (Folke et al., 2010). Within the International Trade Practice context, resilience manifests as the system’s ability to maintain identity and function despite student heterogeneity or changing global trade situations. Through the integration of AI, we have created a more fluid learning environment: the knowledge graph identifies cognitive gaps as they arise, while the synergy between teachers, students, and AI ensures the process stays on track. Instead of a rigid, one-size-fits-all model, this ecological approach allows us to embrace technological change and use it to enhance the way we teach.

### 3.1 Building AI empowered ecological learning environment

**Figure 1:**
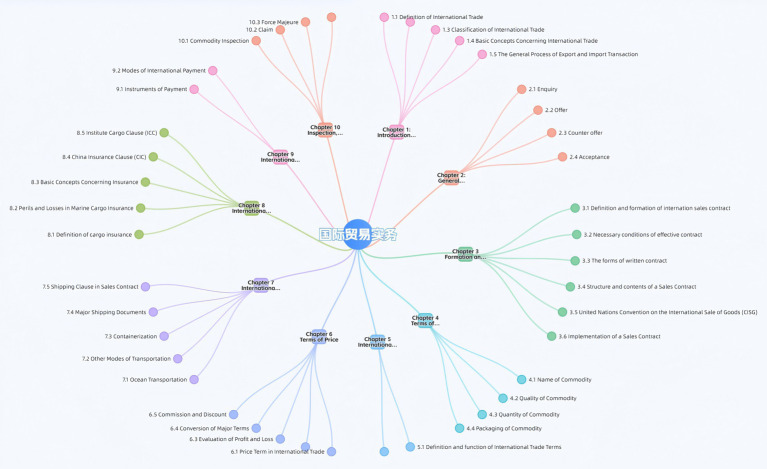
Course knowledge graph of International Trade Practice.

**Figure 2:**
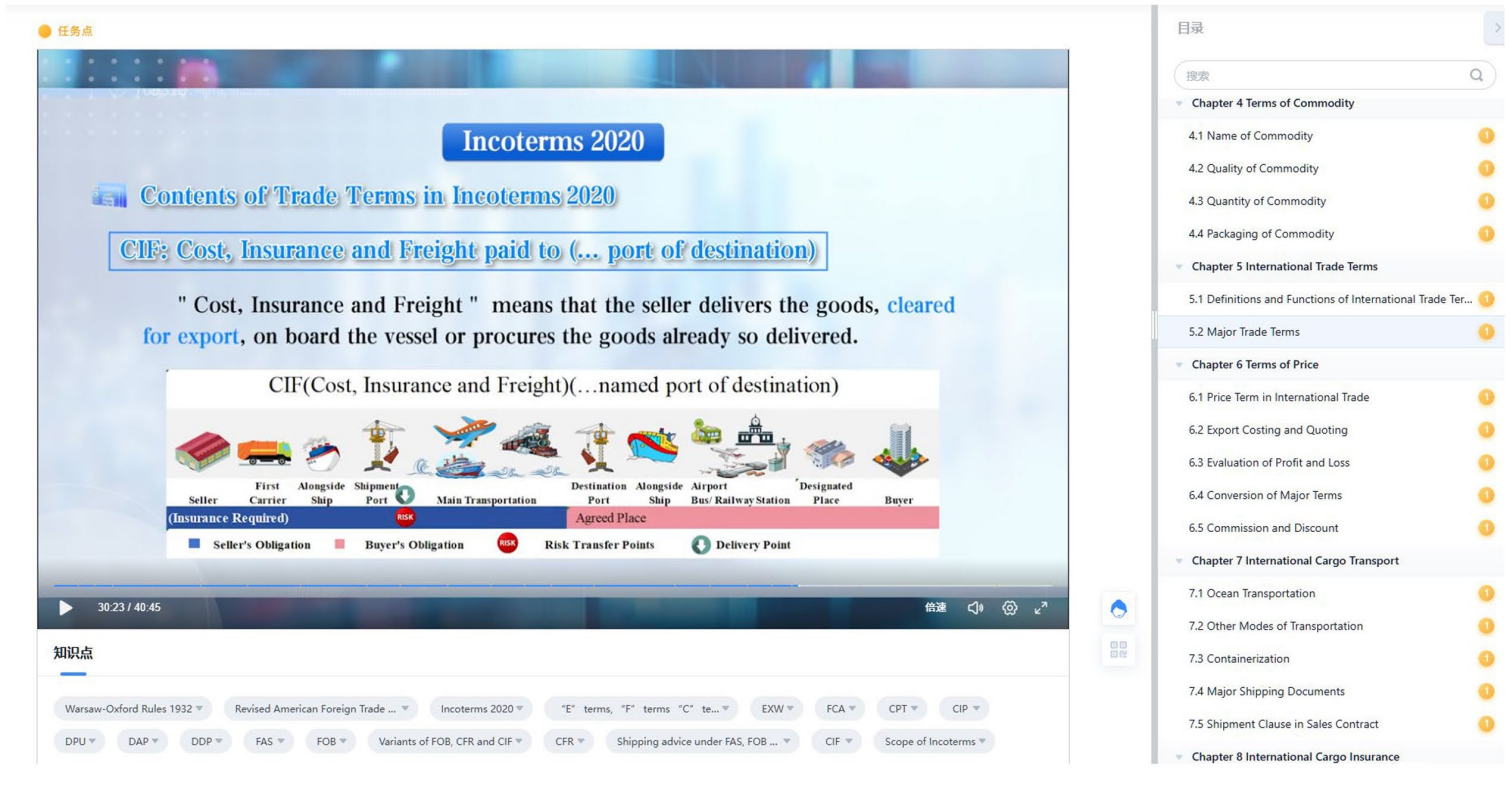
MOOC videos of International Trade Practice.

**Figure 3:**
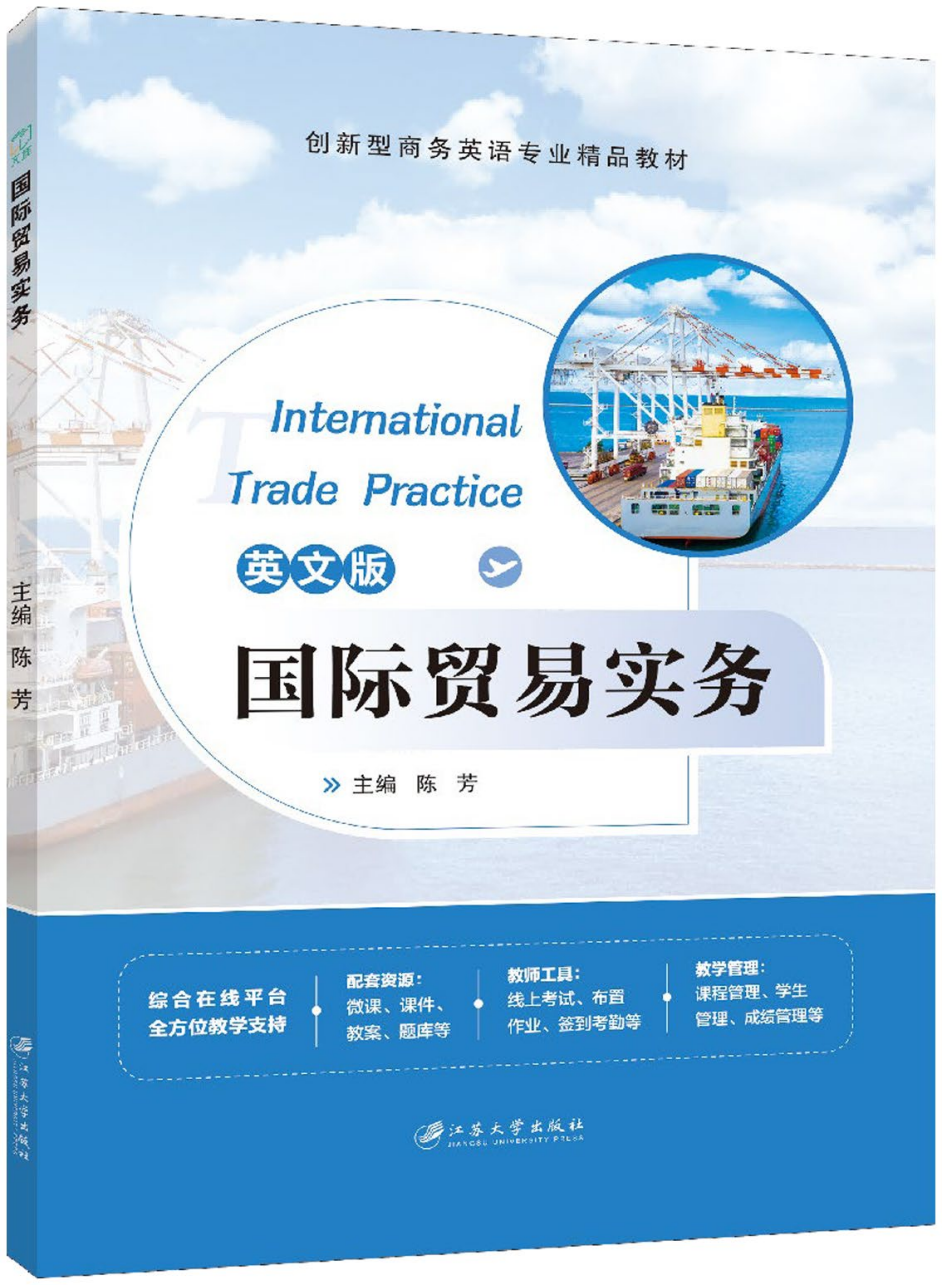
Textbook of International Trade Practice.

To ensure technical rigor, the construction of the knowledge graph followed a structured pipeline of knowledge extraction and semantic modeling. Specifically, named entity recognition and relation extraction algorithms were employed to parse semi-structured data from textbooks and MOOC transcripts, identifying 123 edges and 349 nodes of knowledge points. Subsequently, the course knowledge system was organized to elucidate the logical starting points and core concepts, thereby constructing a systematic disciplinary knowledge graph (Figure 1). Course content tags are used to establish connections between courses, knowledge points, resources, and other related content. The system establishes a proficiency rate for core modules. Students can not progress to the next knowledge point without reaching a 75% mastery threshold in the prerequisite Xueyin knowledge nodes. The objective is to create a smart course graph for the International Trade Practice course that integrates knowledge, skills, and competencies. This will achieve semantic aggregation of educational resources, enable students to search for knowledge and navigate their learning, and provide foundational support for adaptive learning, after-school study, and distance learning. In Hangzhou City University, a structured integration of 43 MOOC videos totaling 463 min (Figure 2), 10 units of courseware, 1 self-compiled textbook (Figure 3), 315 test questions, and various additional resources is implemented through the Xueyin Online Platform. By visualizing these connections through a knowledge graph, students can better understand the overall structure of International Trade Practice and the logical relationships between various knowledge modules.

### 3.2 Shaping the path of the teacher-student-intelligence dynamic interaction

In CLIL programs, AI technology has been utilized to enhance the acquisition of subject knowledge and language proficiency. This is achieved by providing flexible access to learning materials, promoting extensive communication, and facilitating active cooperation throughout the learning process (Sharifi et al., 2018; Tohill, 2007). AI has facilitated the development of the dual-teacher teaching model, where a machine teacher handles auxiliary tasks, such as lesson planning and administration. The “machine teacher” in this study is not a replacement for faculty but a functional redistribution of pedagogical labor. We define the human-machine teacher task allocation matrix based on the complexity and emotional intelligence required for each instructional task (Table 1). Machine teacher can automatically generate context-rich learning materials that are closely linked to specific trade procedures, such as price negotiation, preship inspection, and customs clearance. At the same time, it can track students’ real-time learning progress. The adaptive recommendation engine analyzes student interaction logs, such as dwell time on specific video segments and error patterns in quizzes to calculate mastery rate. When a bottleneck is identified, the system executes a content-based filtering algorithm to push precisely matched remedial resources (e.g., MOOC videos, courseware, book chapters) and Supplementary material (e.g., targeted test items) to ensure the continuity and coherence of the learning process. This immediate intervention ensures that the intelligence functions as a data-driven pedagogical assistant rather than a passive resource repository. In contrast, human teachers focus on core teaching responsibilities, aiming to cultivate students’ foundational competencies and advanced skills. The roles of teachers and students are partially transferred to technology, enabling the digitally reshaping of educational spaces and the driving student learning behaviors and feedback mechanisms.

**Table 1:** The human–machine teacher task allocation matrix.

Task category	Machine teacher’s role	Human teacher’s role
Diagnostic	Real-time tracking of 349 nodes; identifying bottleneck concepts (e.g., 92.59% mastery vs. 43.48% completion).	Interpreting outlier data; identifying systemic gaps in curriculum design.
Evaluative	Automated grading of 315 test items and objective POCIB simulation logs.	Subjective assessment of critical thinking, negotiation ethics, and complex trade disputes.
Instructional	Delivering 463 min of MOOC content; adaptive pushing of remedial resources.	Mentoring; conducting seminars; facilitating simulation debriefs.
Supportive	Providing 24/7 basic query responses via the Xueyin platform.	Providing emotional support and career coaching for struggling students.

**Figure 4:**
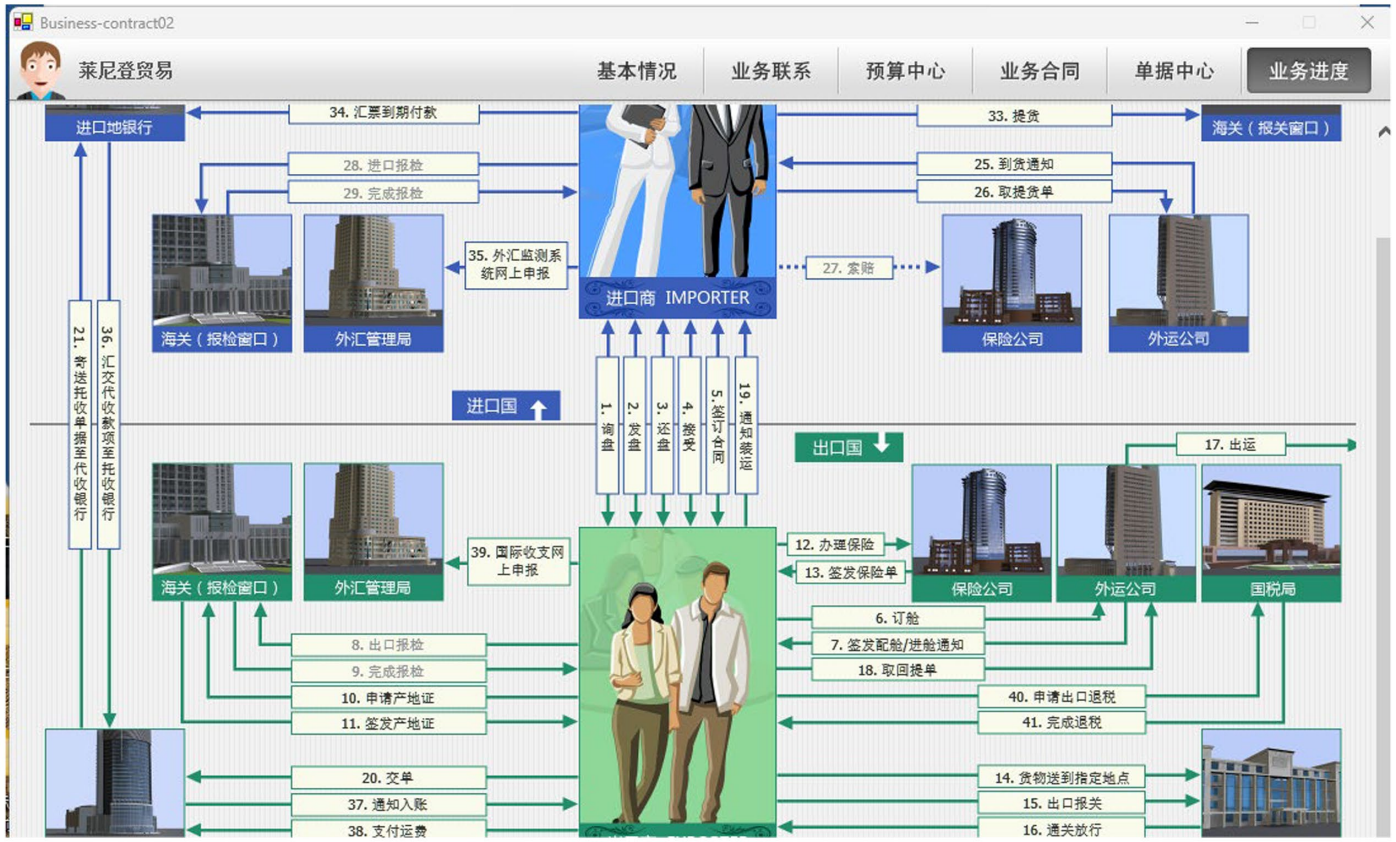
International import and export trade process on POCIB.

Collaborative learning is a vital stage facilitating knowledge construction. Supporting diverse collaborative groups, various collaboration methods, and sustained collaboration time are significant manifestations of the stability and adaptability of a resilient teaching ecosystem (Zhu and Peng, 2020). The POCIB^1^ (Figure 4) simulation software enables learners to work collaboratively in specific experiential scenarios that transcend traditional learning activities (Baek, 2009). This system establishes a risk-free environment to foster experimentation, improve problem-solving capabilities, facilitate evaluation, stimulate social interactions, and serve as a more accessible and immersive experiential learning approach in international business education (Jordan et al., 2024) as well as a tool to support lifelong learning (Baek, 2009).

### 3.3 Constructing the adaptive integration of large-scale teaching and personalization education

**Figure 5:**
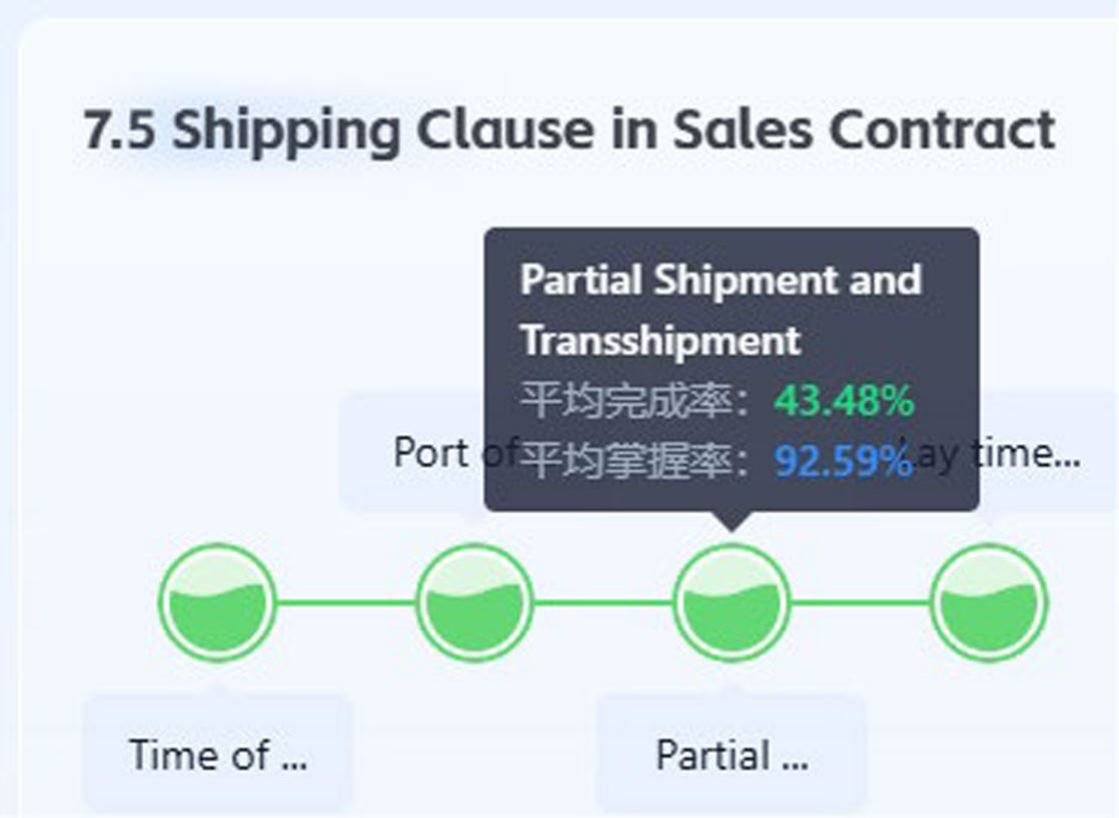
Rates of completion and mastery for the knowledge point of partial shipment and transshipment.

In terms of large-scale teaching, it is essential to leverage the strengths of teachers, enabling them to teach common content and address shared issues. Individualized learning paths are customized based on accurate data from learning evaluations, and learning content is dynamically adjusted to achieve an adaptive knowledge supply and a technologically individualized approach to learning (Edwards and Magill, 2023). The most significant advantage of technology-empowered individualized teaching lies in its adaptability, as the services provided to students are continuously optimized as they evolve. Based on students’ learning behaviors and outcomes (e.g., time, accuracy, and submission attempts for the tests), precise strength and weak point analysis are conducted to facilitate students’ mastery of learning. This allows teachers to transition from a one-size-fits-all approach to data-informed differentiated instruction, while students engage in truly individualized learning. For instance, as illustrated in Figure 5, the mastery rate for the “partial shipment of transshipment” concept reached a high of 92.59%, even though the completion rate was only 43.48%. This discrepancy suggests that students were able to transfer prior knowledge from the “Sales Contract” module in Chapter 3, recognizing the conceptual overlap. Consequently, both instructors and students can bypass redundant material, reallocating their cognitive resources toward more complex or challenging “bottleneck” concepts.

This immediate intervention (Figure 5) confirms that the system functioned as a consistent diagnostic tool. Rather than following a rigid, linear syllabus, the curriculum becomes a living map where knowledge nodes are activated or bypassed based on real-time proficiency. This paradigm shift necessitates a fundamental recalibration of roles within the educational process. The teacher’s role evolves from a primary source of information to a strategic facilitator. With low-level grading and diagnostic tasks automated, instructors can focus on high-value human interactions, such as mentoring, fostering critical thinking, and providing targeted interventions for the small percentage of students who struggle with specific outliers. Furthermore, this framework fosters learner’s sense of academic agency. When students see that their previous knowledge is recognized and that they are not forced to re-learn what they already know, engagement increases and the boredom effect often associated with traditional large-scale teaching is mitigated. Ultimately, such precision facilitates systemic instructional efficacy, enabling institutions to optimize curricula and ensure that pedagogical labor is concentrated where it yields the most significant cognitive gains.

### 3.4 Constructing a holographic smart evaluation method

The scientificity of the evaluation system is the key link to improve the quality of classroom teaching, and also an important part to maintain the vitality of the resilient teaching ecology (Zhai et al., 2021). A resilient educational ecosystem necessitates data-driven intelligent assessment, which is distinguished by its full-process nature, diversification, multi-dimensionality, and visualization. It aims to promote learning and development through evaluation. Based on the CLIL evaluation perspective (Massler et al., 2014) which focuses on the holistic development of students, we established a diversified evaluation system which places emphasis on the all-round development and individualized growth of students to enable a more objective and comprehensive evaluation of students’ learning outcomes, rather than solely concentrating on academic achievements. The formative assessment functions as an all-encompassing system which measures students’ pre-class preparation, in-class participation, and after-class exercises. We utilized big data analysis technologies such as machine learning and deep learning to conduct process-based and multi-dimensional evaluations of students’ learning behaviors, which include the completion rate and replay rate of MOOC videos, the participation rate and accuracy rate in interactive classroom activities, and the accuracy rate of test items on Xueyin Online platform. The final closed book examination serves as a thorough evaluation of students’ high-order synthesis of trade expertise and ESP (English for Specific Purposes) proficiency acquired during classroom sessions. This multi-dimensional evaluation system enables teachers to comprehensively and multidimensionally grasp students’ learning results, focusing on individual differences, thereby providing targeted feedback and guidance. Meanwhile, this system also helps students recognize their strengths and weaknesses, promoting self-improvement.

## 4 The impact of resilient educational ecosystem on learning outcomes: an empirical analysis

### 4.1 Research design

This paper conducts an empirical investigation into the elective course of International Trade Practice that employed a smart course approach to foster a resilient instructional ecosystem throughout the 16-week semester, serving as a pedagogical strategy to yield meaningful outcomes. The study was conducted in a natural educational setting in Hangzhou City University. A quasi-experimental non-equivalent groups design is adopted to contrast the disparities in teaching efficacy between the resilient teaching ecology supported by smart courses and the conventional teaching model. The participants were third-year undergraduate English majors enrolled in the course. They were distributed into two separate teaching classes (*n* = 23 in the CG; *n* = 19 in the EG) through the university’s automated enrollment system. Prior to enrolling in this course, all participants had completed a foundational sequence of business courses delivered through bilingual or English-medium instruction, including Principles of Economics (Bilingual), Fundamentals of Management (English), and International Marketing. This criterion ensured homogeneity in prior content knowledge and ESP exposure, thereby reducing confounding variability in baseline disciplinary literacy. The student’s academic trajectory reflects a deliberate pedagogical design common in Chinese Business English programs, where students first develop general business literacy through foundational courses before advancing to specialized, practice-oriented modules. Consequently, students entered the International Trade Practice course with established familiarity regarding core business terminology, fundamental economic theories, and the cognitive demands of processing disciplinary knowledge through a foreign language. As an elective course offering within the Business English specialization, International Trade Practice attracted students with demonstrated interest in international commerce. The students represented a homogeneous sample in terms of academic standing and disciplinary orientation. While students self-selected the course, they did not self-select their specific teaching class based on the pedagogical intervention, as the smart course-supported model was not disclosed during the enrollment phase. To ensure internal validity, both groups were taught by the same teacher with over 3 years of experience in International Trade concurrently. Since both groups consisted of students who voluntarily elected to take this specialized course, their initial motivation and interest in the subject matter were inherently comparable. We collected data on their prior experience with smart courses. Specifically, 100% of the participants reported experiences of using smart courses on Xueyin platform. Prior to experimentation, a pre-test of the final scores of students from the prerequisite course of Comprehensive English IV confirmed that there were no significant differences in English proficiency between the groups (*p* = 0.75 > 0.05), providing a comparable baseline for the intervention. Consequently, it can be inferred that the criterion for equivalence in the experimental design was met. The study aims to answer two research questions:

(1) Does the resilient educational ecosystem built for the International Trade Practice course yield quantifiable impacts on students’ academic performance?
(2) If this is the case, how does this resilient educational framework shape students’ outcomes in different test items?

The control group (CG, *n* = 23) received traditional education, which involved classroom instruction with a period of 1.5 h per week, supplemented by assigned homework that students needed to complete. The instructional resources given to students in CG were limited to the assigned textbook, unit courseware, and fixed unit tests. The teacher addressed the inquiries mainly through the 15-min tutoring sessions. The smart teaching platform was not used. The experimental group (EG, *n* = 19) implemented a blended teaching approach which merged intelligent course content with their existing educational curriculum. The EG implemented an educational program which encompassed specific interventions of pre-class, in-class and post-class. Before students arrived for the classroom session, the teacher started the class by sharing MOOC videos and relevant cases through the Xueyin Online platform^2^. Students were required to watch the MOOC videos, browse the unit courseware, and finish the instant tests, while the platform delivered diagnostic feedback which guided them to follow personalized learning paths based on the course knowledge graph. During the in-class session, the teacher implemented a 45-min flipped classroom model. As students had acquired the basic disciplinary knowledge through MOOC videos, cases, and diagnosis tests which they completed before class, the teacher focused on addressing the frequently misunderstood knowledge points tracked from pre-class learning data and higher-order cognitive activities such as problem-solving, collaborative practice and in-depth discussions. The teacher has shifted from the role of knowledge transmitters to learning guide. In order to bridge the theory and practice gap, the POCIB platform was incorporated into the class session to simulate the business transaction cycle from establishing business relationships to handling international payments and claims (if possible). Students were guided to set up trade corporations located in different countries assigned by the system randomly. POCIB tracked real-time operational issues through automated node-based diagnostics which assessed students’ proficiency concerning document accuracy, operational speed, budget monitoring abilities. It focused on the closed-loop capabilities of foreign trade business, strengthens the awareness of document processing and compliance, and constructed an in-depth professional preparedness map as well as a individualized learning path for each student, thereby allowing for highly individualized pedagogical modifications. After the class, the Xueyin Online platform pushed tailored supplementary exercises predicated on students’ precious error types. Moreover, the platform generated alerts regarding knowledge mastery and risk warnings to both teacher and students.

**Figure 6:**
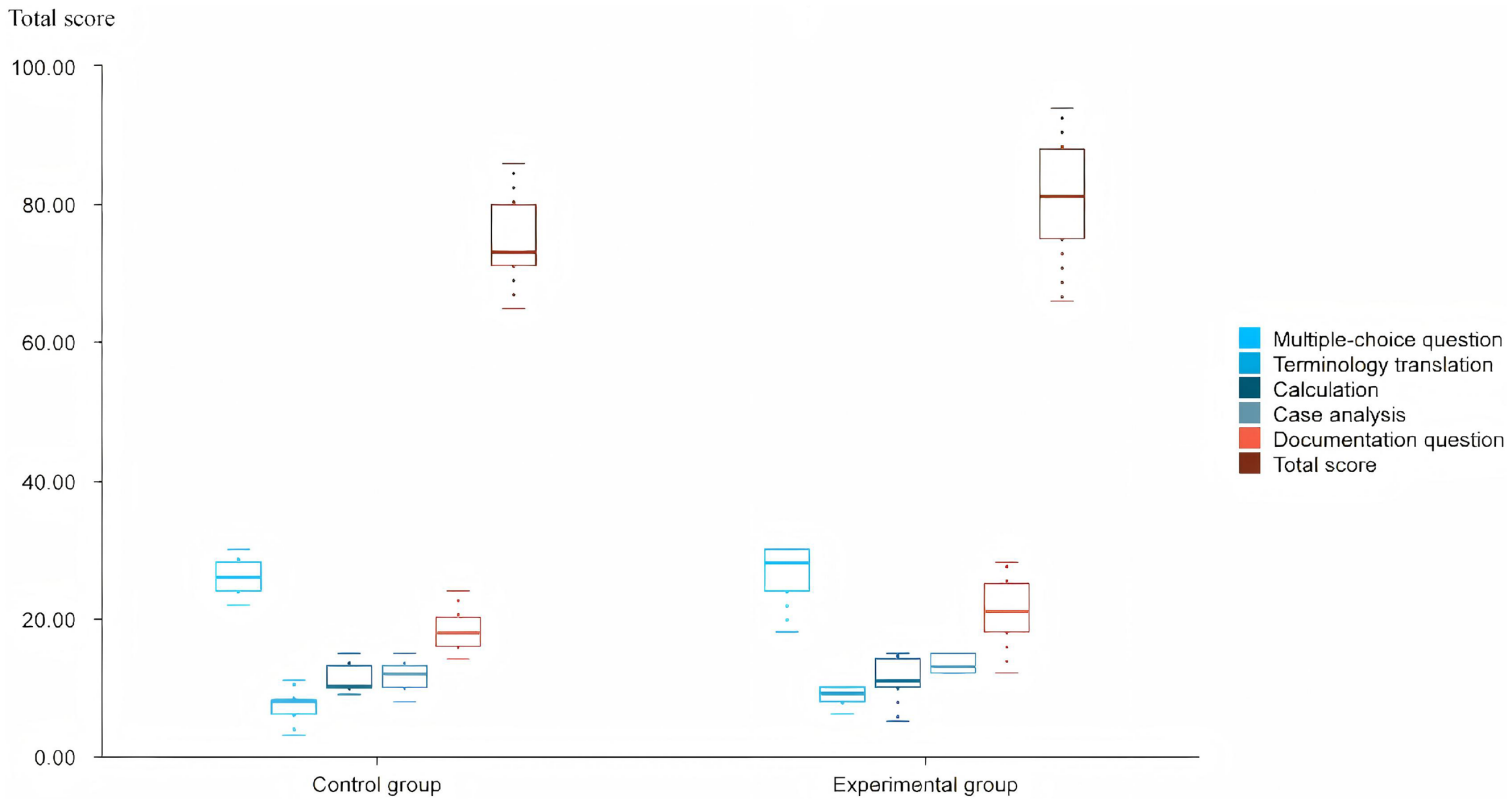
The distribution of student scores of CG and EG.

At the end of the semester, both groups were evaluated using identical, blinded-grading principles for their final outputs, ensuring that the measurement of learning outcomes was free from instructor bias. Both groups participated in a closed-book exam using the same sealed test paper, with a full score of 100 points. The structure and weight of the question types were as follows: multiple-choice questions accounted for 30%, terminology translation questions accounted for 10%, calculation questions accounted for 15%, case analysis questions accounted for 15%, and document questions accounted for 30%. The distribution of student scores is presented in Figure 6.

Regarding the methodology, to assess the actual influence of teaching approaches on enhancing student performance, the SPSSAU software (The SPSSAU Project, 2025) was used to conduct a comparative analysis of performance between the CG and the EG. The research team analyzed the test results by conducting two preceding statistical tests, including Shapiro–Wilk test for data distribution normality and Levene’s test for homogeneity of variance, to ensure the data met the rigorous assumptions required for parametric testing. Based on the outcomes of these two tests, a non-parametric test was subsequently selected to analyze the data between the two groups. Cohen’s d was also employed to quantify the magnitude of the differences between the groups. This method functions properly when the data satisfy the assumptions of normal distribution or homogeneity of variance, enabling a more precise evaluation of the discrepancies (Zhou and Ma, 2024) in the academic performances between two groups. Finally, a post-hoc power analysis was implemented to test the robustness of statistical results of small sample set (Hoenig and Heisey, 2001).

### 4.2 Results and discussion

The normality test functions to identify whether quantitative data from the academic performance of both groups follows a standard normal distribution pattern. The Shapiro–Wilk test functions as an appropriate statistical evaluation because both groups contain fewer than fifty participants (Zhou and Ma, 2024). The data from Table 2 show that the CG exhibited significant differences in the results of the multiple-choice question, terminology translation, calculation, and case analysis (*p* < 0.05), suggesting non-normal distribution pattern. In contrast, the documentation question and the total score displayed no significant differences (*p* > 0.05), which validates the normal distribution data pattern. The EG exhibited significant differences in the results of the multiple-choice questions, terminology translation, and case analysis (*p* < 0.05), confirming the non-normal distribution pattern. On the contrary, the calculation, documentation question, and the total score showed no significant pattern (*p* > 0.05), which validates the normal distribution pattern.

**Table 2:** Result of normality test of CG and EG.

Group	Item	Mean	S.D.	Skewness	Kurtosis	Shapiro–Wilk	
						*W*	*p*-value
CG (*n* = 23)	Multiple-choice question	26.522	2.352	−0.374	−0.578	0.913	0.047*
	Terminology translation	6.783	2.558	−1.877	2.892	0.706	0.000**
	Calculation	11.304	2.162	0.573	−1.222	0.834	0.001**
	Case analysis	11.870	1.890	−0.635	0.090	0.887	0.014*
	Documentation question	18.304	2.670	0.317	−0.515	0.951	0.304
	Total score	74.783	5.705	0.238	−0.928	0.963	0.526
EG (*n* = 19)	Multiple-choice question	26.526	3.518	−1.133	0.662	0.859	0.009**
	Terminology translation	8.737	1.240	−0.612	−0.502	0.868	0.013*
	Calculation	11.316	2.709	−0.393	−0.032	0.939	0.251
	Case analysis	13.526	1.307	0.019	−1.817	0.797	0.001**
	Documentation question	21.474	4.514	−0.416	−0.450	0.953	0.443
	Total score	81.579	8.092	−0.235	−0.772	0.962	0.608

*p < 0.05, **p < 0.01.

The homogeneity of variance test determines whether there are significant disparities in the standard deviation of data fluctuations among groups. As presented in Table 3, the statistical results suggest that the two groups exhibit consistent variability in multiple choice questions, calculation, case analysis, documentation question, and total score (*p* > 0.05), thus meeting the prerequisite for variance analysis (homogeneity of variance), which enables the application of independent-samples t-test to investigate differences. The two groups showed a significant difference (*p* < 0.05) in terminology translation, indicating inconsistent data fluctuations. SPSSAU offers automatic variance adjustment and outputs the adjusted *t*-test results, enabling users to continue referring to the *t*-test results even when confronted with unequal variances (Zhou and Ma, 2024).

**Table 3:** Result of homogeneity of variance (Levene).

Test item	Group type (standard deviation)		*F*	*p*-value
	Control group (*n* = 23)	Experimental group (*n* = 19)		
Multiple-choice question	2.35	3.52	2.482	0.123
Terminology translation	2.56	1.24	4.014	0.052
Calculation	2.16	2.71	0.648	0.426
Case analysis	1.89	1.31	0.569	0.455
Documentation question	2.67	4.51	7.201	0.011*
Total score	5.70	8.09	3.374	0.074

*p < 0.05, **p < 0.01.

What stands out in these results is the notably higher variance in the experimental group (SD = 8.09) compared to the control group (SD = 5.70). This divergence suggests that while the AI-supported pedagogical model significantly raised the class average, it also exerted a differential impact on individual learners. This divergence effect likely stems from disparities in digital literacy and self-regulation. In our resilience framework, it appears that while high-agency students used the AI scaffolding to excel, others may have struggled with cognitive fatigue, widening the achievement gap. Resilient educational ecosystem transforms the divergence from a risk of inequality into an opportunity for personalized learning pathways. This finding underscores the necessity for resilient instructional designs that not only provide advanced AI tools but also offer tiered support to mitigate the digital divide within the classroom.

The non-parametric test is employed to conduct comparative analyses of data differences which follow non-normal distribution patterns or have heterogeneous variance. As indicated in Table 4, the two groups revealed no statistically significant differences in the test items of multiple choice question (*p* > 0.05) and calculation (*p* > 0.05). Conversely, statistically significant differences were identified in areas of terminology translation (*p* < 0.01), case analysis (*p* < 0.01), documentation question (*p* < 0.01), and total score (*p* < 0.01). This outcome indicates that the EG performed well in terms of terminology, case analysis, document questions, and overall scores. The statistical results support the positive impact of the teaching intervention on specific types of test items.

**Table 4:** Result of non-parametric test.

Test Item	Group type median (P_25_, P_75_)		Mann–Whitney test (U)	Mann–Whitney test (*z*)	*p*-value
	CG (*n* = 23)	EG (*n* = 19)			
Multiple-choice question	26.000 (24.0, 28.0)	28.000 (24.0, 30.0)	197.500	−0.546	0.585
Terminology translation	8.000 (6.0, 8.0)	9.000 (8.0, 10.0)	99.500	−3.129	0.002**
Calculation	10.000 (10.0, 13.0)	11.000 (10.0, 14.0)	209.000	−0.245	0.806
Case analysis	12.000 (10.0, 13.0)	13.000 (12.0, 15.0)	116.000	−2.676	0.007**
Documentation question	18.000 (16.0, 20.0)	21.000 (18.0, 25.0)	116.500	−2.595	0.009**
Total score	73.000 (71.0, 80.0)	81.000 (75.0, 88.0)	112.000	−2.695	0.007**

*p < 0.05, **p < 0.01.

We further calculated Cohen’s d as an effect size measure to verify the magnitude of differences between groups. This approach not only reveals the strength of the relationship between variables but also helps determine whether this relationship is of practical significance rather than merely statistically significant. A higher value indicates a greater difference. The benchmarks for identifying small, medium, and large effect sizes are 0.2, 0.5, and 0.8, respectively (Cohen, 1988).

According to Table 5, the Cohen’s d value for the total score is 0.988, which exceeds 0.8, suggesting a moderately large effect. In particular, the Cohen’s d values for terminology translation (0.944), case analysis (1.002), and documentation question (0.876) exceed the threshold of 0.8, indicating a considerable impact. A preliminary inference could be made that the teaching reform leads to a statistically significant improvement in academic achievement and possesses substantial practical implications.

**Table 5:** Result of effect size (in-depth).

Test item	S^2^ pooled (pooled variance)	Cohen’s *d*
Multiple-choice question	8.612	0.002
Terminology translation	4.290	0.944
Calculation	5.874	0.005
Case analysis	2.734	1.002
Documentation question	13.090	0.876
Total score	47.364	0.988

In order to prevent statistical bias or misleading conclusions caused by small sample size, we conducted a post-hoc power analysis using SPSSAU (Table 6). With *α* set at 0.05 and observed means and standard deviations entered as parameters, the analysis yielded a power value of 0.848, which exceeds the conventional threshold of 0.80. This indicates that while the sample size is modest, the pedagogical intervention’s impact was sufficiently robust, thereby enhancing the scientific value and practical application effectiveness of the study. While post-hoc power calculations remain controversial in the literature (Hoenig and Heisey, 2001), we report this value to contextualize our sample size within conventional benchmarks and to inform future replication efforts.

**Table 6:** Results of power analysis.

Alpha (type I error)	Beta (type II error)	Power (1 – beta)	Sample size for group 1 (n1)	Sample size for group 2 (n2)	sample size ratio (n1/n2)
0.05	0.152	0.848	23.000	19.000	1.21053

The experimental group employed a teaching approach which focused on teaching disciplinary knowledge and ESP proficiency through authentic situations and human-computer interaction. This approach facilitates understanding how disciplinary terms functioned dynamically in real work environments while they needed to adapt their communication to different cultural settings. The control group followed conventional teaching methods which placed greater emphasis on teaching unchanging facts, resulting in a relative lack of flexibility in applying knowledge in disciplinary situations. The research results indicate that the teaching reform has exerted significant impacts on enhancing students’ academic performance. Specifically, the teaching reform has brought about improvements which enable students to develop their higher-order cognitive skills represented by the abilities to complete test items such as terminology translation, case analysis, and document questions. The research results confirm previous studies which demonstrate that information and communication technologies can potentially expedite language acquisition and content comprehension by offering different types of learning aids, including visual and auditory support (Martínez-Soto and Prendes-Espinosa, 2023; Diezmas, 2018). On the other hand, no significant difference was observed between the test items of multiple choice question and calculation. This phenomenon can be explained by the fact that fundamental computation mainly assesses memory and simple arithmetic abilities, which can be acquired through continuous reinforcement rather than innovative reforms. The standardized instructions and tasks utilized within the CG proved adequate for successfully teaching computational principles. Conversely, the novel pedagogical strategies implemented in the EG did not yield supplementary improvements.

When teaching reforms prioritize more higher-order cognitive abilities like analyzing real cases, they fail to create substantial effects on more foundational abilities. From the perspective of educational theory, these research results align with the enhancement of the “understanding and application” levels in Bloom’s taxonomy of cognitive objectives (Bloom, 1956; Anderson and Krathwohl, 2001). The findings reinforce the specific functions of teaching reforms in enhancing linguistic and professional proficiency in International Trade Practice, thus providing empirical substantiation for subsequent course optimization in the intelligent era. Nevertheless, this interpretation is predicated on inferences derived from inter-group statistical data in a naturalistic educational setting. The underlying rationales need to be further verified by controlling more baseline variables (i.e., prior international trade knowledge, digital literacy) and incorporating teaching logs, student interviews, or intervention details to mitigate the impact of sampling bias or random factors and strengthen causal claims.

The research findings also suggest that instructional design should be optimized based on the features of different test items. For fundamental test items, traditional teaching methods can be upheld, but efforts should be focused on developing more advanced skills. For example, incorporating international trade scenario simulations into calculations may enhance the strengths observed in the experimental group. Moreover, attention must be given to sample diversity (e.g., disparities in individual students, calculation proficiency) that might obscure subtle effects.

To summarize, the results of quasi-experimental research demonstrate that the main advantages of the construction of a resilient instruction ecology of the CLIL course lie in the fact that learners acquire specific literacy practices linked to the curricular subjects they study via the language. These proficiencies exceed what is typically acquired in a foreign language curriculum (Hüttner and Dalton-Puffer, 2024). This aligns with the idea that innovative teaching approaches, potentially involving interactive or practical components, enhance student learning outcomes. Practically, the findings provide support for instructors, recommending the promotion of such innovative reform methods in comparable courses to lessen inter-university disparities and enhance teaching quality.

## 5 Conclusion

This study leverages AI technology to form a resilient educational ecosystem by constructing a smart course, exploring innovative teaching models, and reshaping human-machine collaborative CLIL teaching scenarios in the context of digital intelligence, thereby addressing students’ knowledge disorientation and information overload. The empirical study results indicate that the EG performed significantly better than the CG in test items of terminology translation, case analysis, document questions, and total scores. The research findings support the positive impact of teaching reform on specific types of test items, providing empirical evidence for subsequent course optimization. The resilient educational ecosystem appears to foster metacognitive readiness, providing students with the digital scaffolding necessary for future autonomous learning. It adapts to the development needs of future society and has important enlightenment for global education reform and development. Moreover, this ecosystem provides a practical approach for implementing policies in China, such as the “Education Modernization 2035” (Ministry of Education, 2019) and “Four Futures” (Ministry of Education, 2025).

The findings of this study are limited to some extent. First, the pretest relied solely on language proficiency, which provides an incomplete picture of the students’ baseline in international trade. This leaves room for selection bias, as pre-existing differences in student motivation or technical proficiency might have influenced the outcomes. To achieve more robust causal inference, future studies should implement a more comprehensive suite of pre-intervention assessments. Second, limited by its single-point assessment, the study provides evidence of near-transfer within the pedagogical framework, precluding causal inferences about longitudinal skill development. Future longitudinal research is required to track the persistence of these skills and their far-transfer to diverse professional contexts beyond the classroom. Third, the small sample size from the single-institution, single-course limits external validity. To enhance the generalizability and robustness of the findings, future research should consider expanding the sample size, extending the evaluation period, or incorporating additional evaluation dimensions to further verify statistical significance. Fourth, despite these promising system-wide outcomes, the overlapping nature of the interventions makes it difficult to isolate the exact impact of any single feature. Our future work will focus on unbundling these factors, perhaps through more discrete trials, to better understand what truly drives the efficacy we observed.

## Data Availability

The original contributions presented in the study are included in the article/Supplementary material, further inquiries can be directed to the corresponding author.

## Footnotes

1 The software of Professional Operations Competency in International Business (POCIB). It provides a simulated environment to comprehensively develop practical international trade operational skills, enhance professional competencies, and support career development.

2 https://www.xueyinonline.com/.Itis a innovative open learning MOOC platform which operates high-quality online courses for higher education, vocational training, and lifelong learning in China.
